# TMEM39A acts as a novel profibrotic factor to activate hepatic stellate cells partially via the cell adhesion molecule signaling axis

**DOI:** 10.3389/fcell.2026.1926987

**Published:** 2026-09-28

**Authors:** Meng Kong, Lihong Zhang, Yuexia Bai, Jinhua Jia, Chuanyang Liu, Wei Liu, Shuai Chen, Shisong Zhang

**Affiliations:** 1 Department of Pediatric Surgery, Children’s Hospital Affiliated to Shandong University, Jinan, China; 2 Department of Pediatric Surgery, Jinan Children’s Hospital, Jinan, China; 3 Department of Pathology, Children’s Hospital Affiliated to Shandong University, Jinan, China

**Keywords:** cell adhesion molecules, hepatic stellate cells, ICAM-1, liver fibrosis, TMEM39A

## Abstract

**Objective:**

Hepatic stellate cell (HSC) transdifferentiation into myofibroblasts is a pivotal event in liver fibrosis, yet the underlying regulatory network remains elusive. This study explores the role of TMEM39A in HSC activation and its pro-fibrotic effects via the cell adhesion molecule (CAM) signaling axis.

**Methods:**

An *in vitro* HSC activation model was established using TGF-β1-stimulated LX-2 cells, combined with TMEM39A knockdown and overexpression. Cell viability, migration, and apoptosis were assessed by CCK-8, wound healing, Transwell, and flow cytometry. Fibrotic marker expression was measured at both mRNA and protein levels. RNA sequencing and bioinformatics identified the CAM pathway as a candidate downstream effector, and its pharmacological inhibitor (Alicaforsen, an ICAM-1 antagonist) was used for validation.

**Results:**

TMEM39A expression correlated positively with HSC activation. Overexpression of TMEM39A promoted LX-2 viability, migration, and upregulation of fibrotic markers (α-SMA, COL1A1) while suppressing apoptosis, whereas knockdown reversed these effects. Mechanistically, TMEM39A activated the CAM axis with specific ICAM-1 elevation. Alicaforsen treatment partially inhibited the activation of HSCs and the expression of fibrosis markers induced by TMEM39A.

**Conclusion:**

TMEM39A functions as a novel pro-fibrotic facilitator that promotes HSC activation at least partially through the CAM/ICAM-1 signaling axis. Our findings provide mechanistic insights into liver fibrosis and suggest TMEM39A and its downstream effectors as potential candidates for further therapeutic exploration, pending rigorous *in vivo* and clinical validation.

## 1 Introduction

Liver fibrosis is a common pathological process underlying the progression of various chronic liver diseases toward cirrhosis and hepatocellular carcinoma, yet effective anti-fibrotic therapies remain scarce. Persistent chronic liver injury—resulting from conditions such as biliary atresia, viral hepatitis, alcohol abuse, and metabolic disorders—triggers the transdifferentiation of quiescent HSCs into activated myofibroblasts, a core event driving the initiation and progression of liver fibrosis (Kong et al., 2026; Higashi et al., 2017; Khomich et al., 2019). Activated HSCs are the principal source of extracellular matrix (ECM) proteins, particularly collagens, in the liver; their excessive deposition, coupled with impaired degradation, constitutes the pathological basis of fibrosis (Ruan et al., 2019; Li et al., 2018). HSC activation is governed by complex signaling networks, including the transforming growth factor-β (TGF-β), tumor necrosis factor-α (TNFα), and various non-coding RNAs (Zhang et al., 2024; Kitano and Bloomston, 2016; Yang and Seki, 2015). Recent advances have highlighted the involvement of epigenetic regulation, metabolic reprogramming, and cellular senescence in HSC activation, offering new therapeutic avenues (El Taghdouini and van Grunsven, 2016; Lee et al., 2025; Serna-Salas et al., 2023). Nevertheless, owing to the complexity and heterogeneity of liver fibrosis pathogenesis, no clinically approved drug can effectively reverse or halt fibrosis progression.

Under physiological conditions, HSCs remain quiescent, storing vitamin A-laden lipid droplets. Upon chronic injury, however, they become activated, transdifferentiating into myofibroblast-like cells that acquire pro-fibrotic, proliferative, migratory, and anti-apoptotic properties, while secreting large amounts of ECM components, thereby disrupting liver structure and function (Yu et al., 2015; Wang et al., 2015). Elucidating the molecular mechanisms governing HSC activation and their key biological behaviors—proliferation, migration, and resistance to apoptosis—remains a central challenge in fibrosis research. Multiple signaling pathways and molecules, including the TGF-β/Smad, PTEN/PI3K/AKT pathways, and various microRNAs, have been implicated in regulating HSC activation (Zhang et al., 2026; Hao et al., 2018). For instance, miR-17–5p promotes HSC proliferation and activation by targeting Smad7 (Yu et al., 2015), whereas the histone deacetylase inhibitor givinostat suppresses HSC proliferation and induces apoptosis via modulation of NF-κB and SOD2 acetylation (Wang et al., 2015). Similarly, miR-654–5p enhances HSC proliferation and inhibits apoptosis by targeting RXRα (Ma et al., 2022), and CAT1 promotes HSC activation and fibrosis through IL-6-mediated inflammatory pathways (Deng et al., 2026). Despite these insights, the regulatory network governing HSC activation remains incompletely understood, and identifying novel key regulators is crucial for developing effective anti-fibrotic strategies.

Genome-wide association studies (GWAS) initially linked a single nucleotide polymorphism (rs1132200) of transmembrane protein 39A (TMEM39A) to multiple sclerosis risk, suggesting its involvement in central nervous system inflammation (International Multiple Sclerosis Genetics Consortium IMSGC, 2010). Subsequent studies identified TMEM39A alleles in the chromosome 3q13.33 region as susceptibility loci for primary biliary cholangitis, an autoimmune liver disease (Hitomi et al., 2019). Moreover, epigenetic studies on Alzheimer’s disease revealed sex-specific differences in TMEM39A DNA methylation, with the gene closely associated with immune processes such as inflammation and dysregulated type I interferon responses (Zhang L. et al., 2021). In oncology, a synthetic peptide derived from TMEM39A (TMEM39AS41) was shown to induce cell death in KRAS-mutant lung cancer cells by inhibiting the inflammation/autophagy pathway and to suppress tumor growth in mice (Park et al., 2020). Collectively, these findings highlight the critical roles of TMEM39A in inflammatory responses, immune regulation, and cancer progression.

Although GWAS have linked the 3q13.33 region (containing TMEM39A) to genetic susceptibility to autoimmune liver diseases such as primary biliary cholangitis, suggesting its potential involvement in liver pathology (Hitomi et al., 2019), its specific biological function in liver fibrosis—particularly in HSC activation and downstream signaling—has not been reported. TMEM39A is an ER-resident transmembrane protein whose nematode ortholog (TMEM-39) coordinates with COPII vesicle complexes to regulate collagen secretion and ER stress responses (Sung et al., 2025). In mammalian cells, TMEM39A has been implicated in autophagy modulation and viral replication, but its role in hepatic fibrosis has not been explored. Current knowledge of TMEM39A remains largely confined to genetic associations, with limited mechanistic exploration in liver fibrosis. To address this gap, the present study systematically evaluated the regulatory effects of TMEM39A on HSC proliferation, migration, apoptosis, and fibrosis marker expression using a TGF-β1-induced LX-2 cell activation model, employing both gain- and loss-of-function approaches. Our aim was to uncover the role and molecular mechanisms of TMEM39A in HSC activation and to provide new theoretical evidence for targeted anti-fibrotic intervention.

Transcriptome sequencing and bioinformatics analysis suggested that TMEM39A may exert its pro-fibrotic effects through the CAM pathway. To validate this finding and elucidate the underlying mechanism, we selected ICAM-1, a key CAM pathway molecule, as a representative target and used the specific small-molecule inhibitor Alicaforsen in functional rescue experiments. We assessed whether pathway inhibition could reverse the HSC activation and fibrotic phenotype induced by TMEM39A overexpression. Our results demonstrated that CAM pathway blockade significantly attenuated TMEM39A-induced upregulation of activation and fibrosis markers, confirming that TMEM39A relies, at least in part, on this pathway to exert its pro-fibrotic effects. Thus, targeting TMEM39A and its downstream CAM pathway represents a promising novel strategy for liver fibrosis treatment.

## 2 Materials and methods

### 2.1 Cell culture and treatment

The human hepatic stellate cell line LX-2 was obtained from the American Type Culture Collection (ATCC). Cells were cultured in high-glucose Dulbecco’s modified Eagle’s medium (DMEM, Gibco, United States) supplemented with 10% fetal bovine serum (FBS) and 1% penicillin/streptomycin in a humidified incubator at 37 °C with 5% CO_2_. To induce HSC activation, LX-2 cells were treated with recombinant human TGF-β1 (No. CA59; Novoprotein, China) at a final concentration of 10 ng/mL for 24 h (Kong and Xiang, 2021).

### 2.2 Cell transfection and model construction

Small interfering RNA targeting human TMEM39A (si-TMEM39A) and its negative control (si-NC), as well as a plasmid carrying the full-length coding sequence of TMEM39A (oe-TMEM39A) and the corresponding empty vector (oe-NC), were designed and synthesized by Shanghai GenePharma Co., Ltd. LX-2 cells at 70%–80% confluence were transiently transfected using Lipofectamine 8000 (No. C0533; Beyotime, China) according to the manufacturer’s instructions. Six hours post-transfection, the medium was replaced with complete medium, and cells were cultured for an additional 48 h before harvesting. Transfection efficiency was verified by RT-qPCR and Western blotting.

### 2.3 RNA extraction and real-time quantitative PCR (RT-qPCR)

Total RNA was extracted from LX-2 cells using TRIzol® reagent. RNA concentration and purity were determined with a Nanodrop 2000 spectrophotometer. One microgram of total RNA was reverse-transcribed into cDNA using HiScript® III RT SuperMix. RT-qPCR was performed using ChamQ Universal SYBR qPCR Master Mix on an ABI StepOnePlus™ Real-Time PCR System. GAPDH served as an internal control, and relative gene expression was calculated using the 2^−^ΔΔCt method. Primer sequences were synthesized by Beijing Tsingke Biotech Co., Ltd. (Supplementary Table S1).

### 2.4 Western blotting

Total protein was extracted using RIPA lysis buffer (No. P0013B; Beyotime, China) containing protease and phosphatase inhibitors, and protein concentration was determined using the BCA method (No. P0011; Beyotime, China). Equal amounts of protein (20–30 μg) were separated by SDS-PAGE and wet-transferred onto PVDF membranes (No. FFP39; Beyotime, China). After blocking with 5% non-fat milk for 1 h at room temperature, membranes were incubated overnight at 4 °C with primary antibodies: rabbit anti-human TMEM39A polyclonal (20430-1-AP, Proteintech), rabbit anti-human α-SMA (ab124964,Abcam), rabbit anti-human COL1A1 (ab316222, Abcam), and rabbit anti-human ICAM-1 (ab282575, Abcam). After TBST washing, membranes were incubated with HRP-conjugated goat anti-rabbit secondary antibody (ab6721, Abcam) for 1 h at room temperature. Protein bands were visualized using enhanced chemiluminescence (ECL) reagent (No. P0018S, Beyotime), and semi-quantitative analysis of band intensities was performed using ImageJ software.

### 2.5 Cell viability assay (CCK-8)

Transfected LX-2 cells were seeded into 96-well plates at 3 × 10^3^ cells per well (5 replicates per group). At 24, 48, 72, and 96 h, 10 μL of CCK-8 solution (No. C0037; Beyotime) was added to each well and incubated at 37 °C for 2 h in the dark. Absorbance at 450 nm was measured using a microplate reader.

### 2.6 Cell migration assays

Wound healing assay: Transfected LX-2 cells were seeded into 6-well plates. At 100% confluence, a straight scratch was made using a 200-μL sterile pipette tip. After PBS washing, the medium was replaced with low-serum DMEM (0.5% FBS). Images were captured at 0 and 48 h, and the wound area was measured using ImageJ to calculate the healing rate.

Transwell migration assay: Transfected cells were resuspended in serum-free medium at 2 × 10^5^ cells/mL, and 200 μL of the suspension was added to the upper chamber of Transwell inserts (8-μm pore size; No. 3422, Kimble, United States). The lower chamber contained 600 μL of DMEM with 30% FBS as a chemoattractant. After 24 h, non-migrated cells on the upper surface were removed, and migrated cells on the lower surface were fixed with 4% paraformaldehyde and stained with 0.1% crystal violet. Five random fields (×200) were counted under a light microscope.

### 2.7 Apoptosis assay

Apoptosis was assessed using the Annexin V-FITC/PI Apoptosis Detection Kit (Bioscience, Shanghai, China). Transfected LX-2 cells cultured for 48 h were harvested, washed with PBS, and resuspended in 500 μL of Binding Buffer. Then, 5 μL of Annexin V-FITC and 10 μL of PI were added and incubated for 15 min at room temperature in the dark. Apoptosis was analyzed by flow cytometry, and data were processed using FlowJo software.

### 2.8 Transcriptome sequencing (RNA-seq) and bioinformatics analysis

LX-2 cells from the oe-TMEM39A and oe-NC groups were collected for total RNA extraction. It should be noted that the three biological replicate samples are independent cell culture passages. After quality control, paired-end sequencing was performed on an Illumina NovaSeq 6000 platform by Wuhan Seqhealth Technology Co., Ltd. Differentially expressed genes (DEGs) were identified using DESeq2 (|log2FC| > 1, *P* < 0.05). GO functional and KEGG pathway enrichment analyses were performed on DEGs. GSEA was used to assess overall expression trends of gene sets related to specific pathways.

### 2.9 Rescue experiments

Based on RNA-seq results, we selected the CAM pathway and used the ICAM-1-specific inhibitor Alicaforsen for functional rescue. LX-2 cells were divided into three groups: control (oe-NC), TMEM39A overexpression (oe-TMEM39A), and rescue (oe-TMEM39A+ Alicaforsen). After transfection, cells were treated with 10 μM Alicaforsen for 48 h (Supplementary Figure S1). Expression levels of ICAM-1, α-SMA, and COL1A1 were detected by RT-qPCR and Western blotting.

### 2.10 Statistical analysis

All experiments were independently repeated at least three times. Data are presented as mean ± standard deviation (SD). Statistical analyses were performed using SPSS 26.0 (Chicago, IL, United States) and GraphPad Prism 9.0 (California, United States). Comparisons between two groups were made using unpaired Student’s t-test; multiple-group comparisons were performed using one-way ANOVA. A P value <0.05 was considered statistically significant.

## 3 Results

### 3.1 TMEM39 A promotes TGF-β1-induced LX-2 cell activation and fibrotic marker expression

To investigate the role of TMEM39A in HSC activation, LX-2 cells were stimulated with 10 ng/mL TGF-β1, which significantly upregulated α-SMA and COL1A1 mRNA expression (Figure 1A). In this model, si-TMEM39A knockdown significantly suppressed TGF-β1-induced α-SMA and COL1A1 upregulation, whereas oe-TMEM39A overexpression further enhanced their expression compared with respective controls (Figures 1B,C; Supplementary Figure S2). These results indicate that TMEM39A is upregulated in activated LX-2 cells and promotes HSC activation by modulating α-SMA and COL1A1 expression, suggesting TMEM39A as a key regulatory factor and potential therapeutic target in liver fibrosis.

**FIGURE 1:**
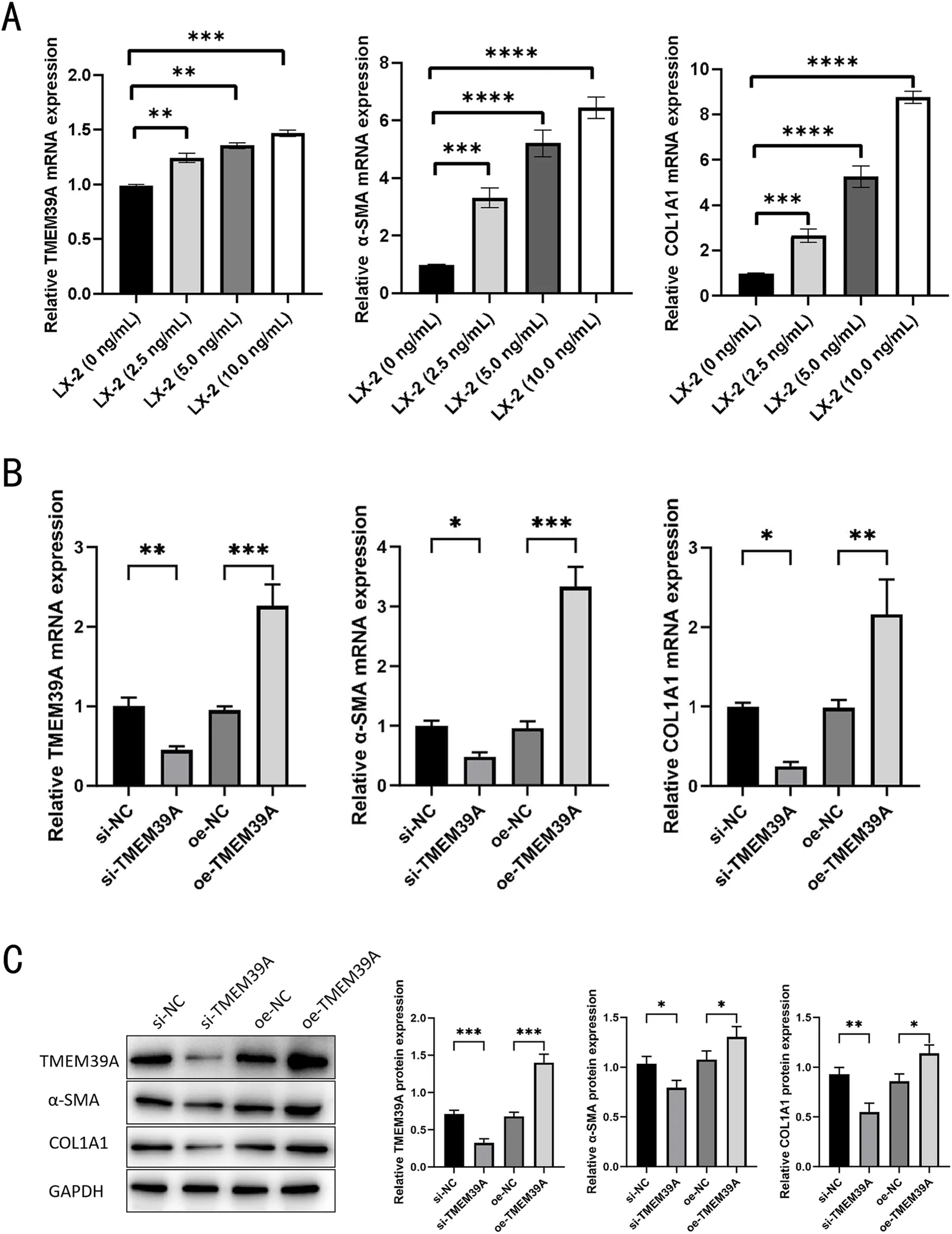
TMEM39A promotes LX-2 HSC activation and upregulates fibrosis-related genes α-SMA and COL1A1. **(A)** Dose-dependent activation assay: LX-2 cells treated with increasing TGF-β1 concentrations (0, 2.5, 5.0, 10.0 ng/mL) showed dose-dependent upregulation of TMEM39A, α-SMA, and COL1A1 mRNA, indicating a positive correlation between TMEM39A expression and HSC activation status. **(B)** mRNA levels after siRNA knockdown (si-TMEM39A) or plasmid overexpression (oe-TMEM39A): compared with controls, si-TMEM39A reduced TMEM39A, α-SMA, and COL1A1 expression, whereas oe-TMEM39A increased them. **(C)** Protein levels by Western blotting: consistent with mRNA trends, TMEM39A knockdown decreased, and overexpression increased, α-SMA and COL1A1 protein levels, confirming its positive regulatory role in the fibrotic phenotype. *P < 0.05, **P < 0.01, ***P < 0.001, ***P < 0.0001.

### 3.2 TMEM39A promotes LX-2 cell viability and migration and inhibits apoptosis

CCK-8 assays showed that TMEM39A knockdown significantly reduced LX-2 cell viability at 48, 72, and 96 h, whereas overexpression significantly enhanced cell viability (Figure 2A). Wound healing and Transwell assays revealed that TMEM39A knockdown decreased, while overexpression increased, cell migration (Figures 2B,C). Flow cytometry demonstrated that TMEM39A knockdown increased apoptosis rates, whereas overexpression decreased them (Figure 2D). Collectively, these results demonstrate that TMEM39A promotes HSC viability and migration while exerting an anti-apoptotic effect.

**FIGURE 2:**
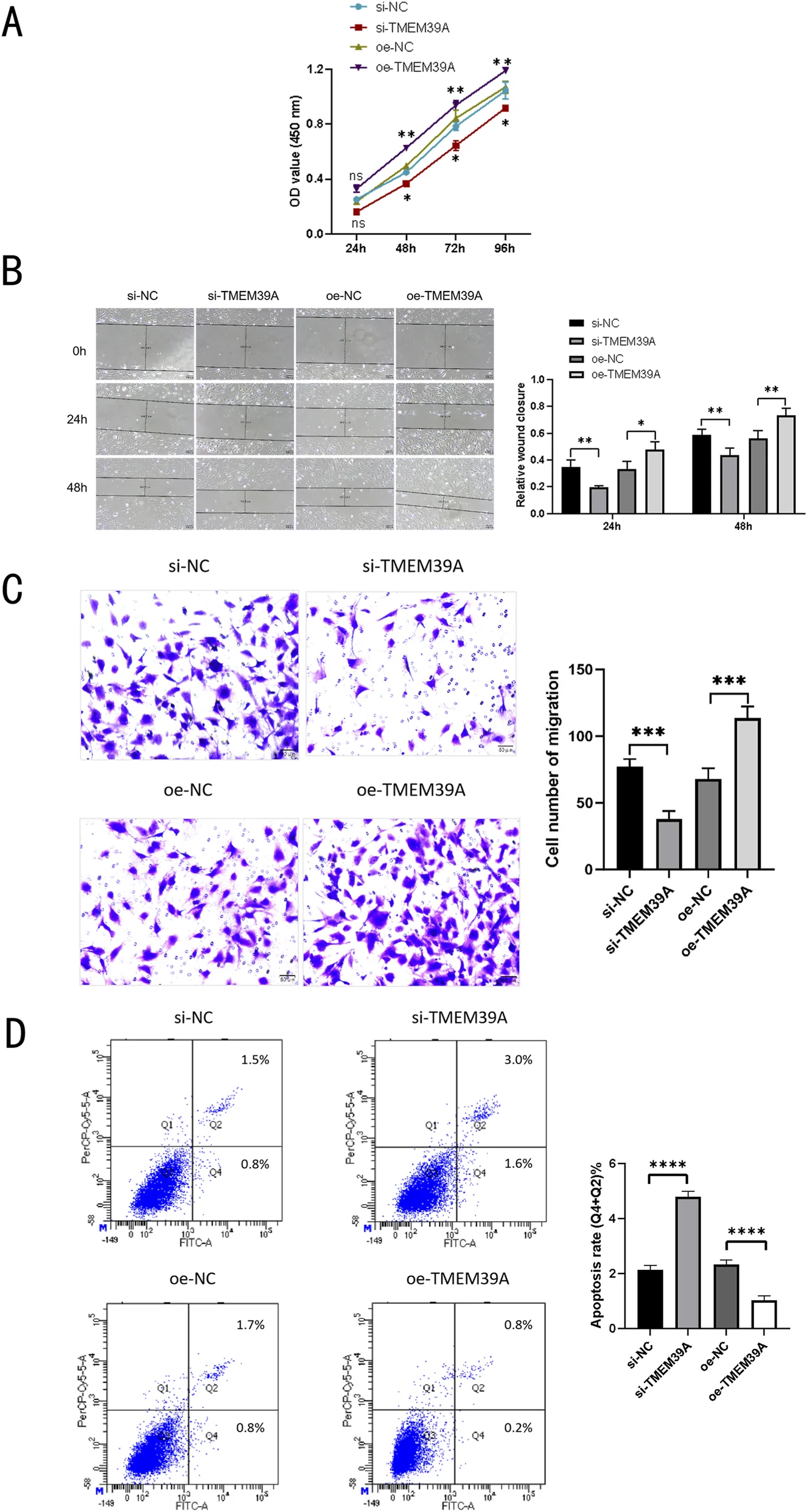
TMEM39A promotes LX-2 cell viability and migration and inhibits apoptosis. **(A)** CCK-8 assay showing OD 450 nm values at 24, 48, 72, and 96 h after TMEM39A overexpression or knockdown. **(B)** Wound healing assay showing migratory ability at 24 and 48 h. **(C)** Transwell migration assay showing transmigratory capacity. **(D)** Flow cytometry (Annexin V-FITC/PI) showing apoptosis rates. *P < 0.05, **P < 0.01, ***P < 0.001, ***P < 0.0001.

### 3.3 Transcriptome sequencing reveals potential involvement of the cell adhesion molecule pathway

PCA revealed distinct clustering of oe-TMEM39A and oe-NC samples, Sample correlation coefficients within each group exceeded 0.82, confirming high reproducibility among biological replicates (Supplementary Figures S3–S5). RNA-seq analysis identified 750 DEGs between oe-TMEM39A and oe-NC groups (|log2FC| > 1, *P* < 0.05), including 652 upregulated and 98 downregulated genes (Figures 3A,B; Supplementary Tables S2-S3). GO analysis revealed enrichment in cell-cell adhesion, cell-matrix adhesion, and cell migration (Figure 3C). KEGG analysis highlighted pathways including adherens junction, MAPK signaling, and autophagy (Figure 3D). GSEA confirmed significant positive enrichment of adherens junction organization (GO:0034332) in the oe-TMEM39A group (NES = 1.828, *P* = 0.000, FDR = 0.035) (Figure 3E). A heatmap of key genes in the adherens junction pathway showed general upregulation in oe-TMEM39A cells (Figure 3F). These results indicate that TMEM39A may promote HSC activation and fibrosis through broad effects on cell adhesion- and migration-related genes, particularly via the CAM pathway.

**FIGURE 3:**
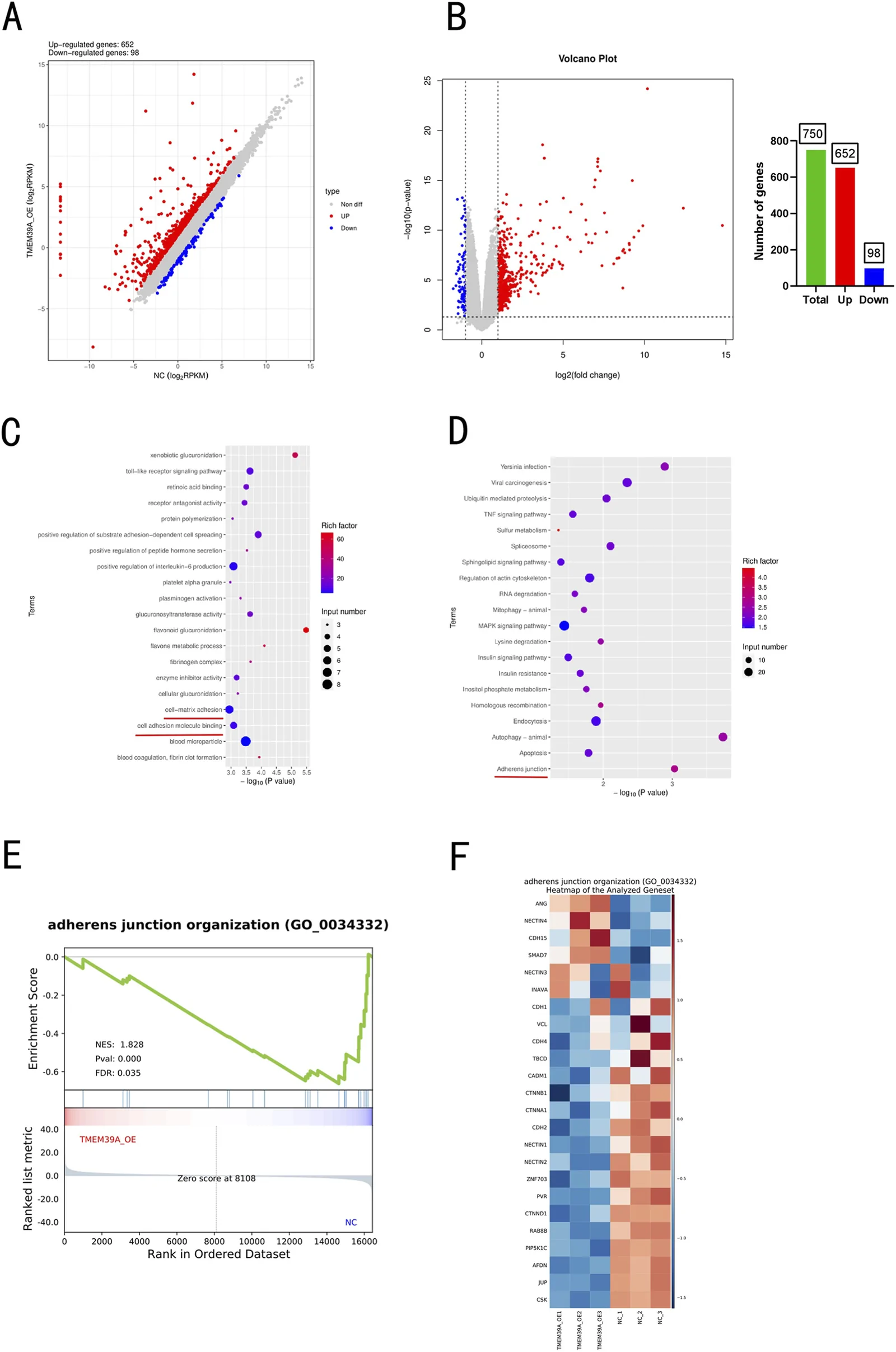
Transcriptomic effects of TMEM39A overexpression and GSEA analysis. **(A)** Scatter plot of DEGs between oe-TMEM39A and oe-NC groups (red: upregulated; blue: downregulated). **(B)** Volcano plot of DEGs based on log2FC and–log10(P-value). **(C)** GO biological process enrichment bubble plot. **(D)** KEGG pathway enrichment bubble plot. **(E)** GSEA showing significant enrichment of adherens junction organization (GO:0034332) in oe-TMEM39A group (NES = 1.828, FDR = 0.035). **(F)** Heatmap of core DEGs in the cell adhesion junction pathway.

### 3.4 Rescue experiments confirm TMEM39A promotes fibrosis through the CAM pathway

To validate the downstream mechanism, we focused on the CAM pathway and used the ICAM-1 inhibitor Alicaforsen. RT-qPCR showed that oe-TMEM39A significantly upregulated ICAM-1, α-SMA, and COL1A1 mRNA levels, which were partially reversed by Alicaforsen treatment (Figure 4A). Western blotting confirmed similar changes at the protein level (Figure 4B; Supplementary Figure S2). These findings demonstrate that Alicaforsen effectively counteracts TMEM39A overexpression-induced HSC activation, confirming that the CAM pathway—mediated by ICAM-1—is a critical downstream effector of TMEM39A in promoting liver fibrosis. Moreover, Alicaforsen may represent a potential candidate for anti-fibrotic therapy.

**FIGURE 4:**
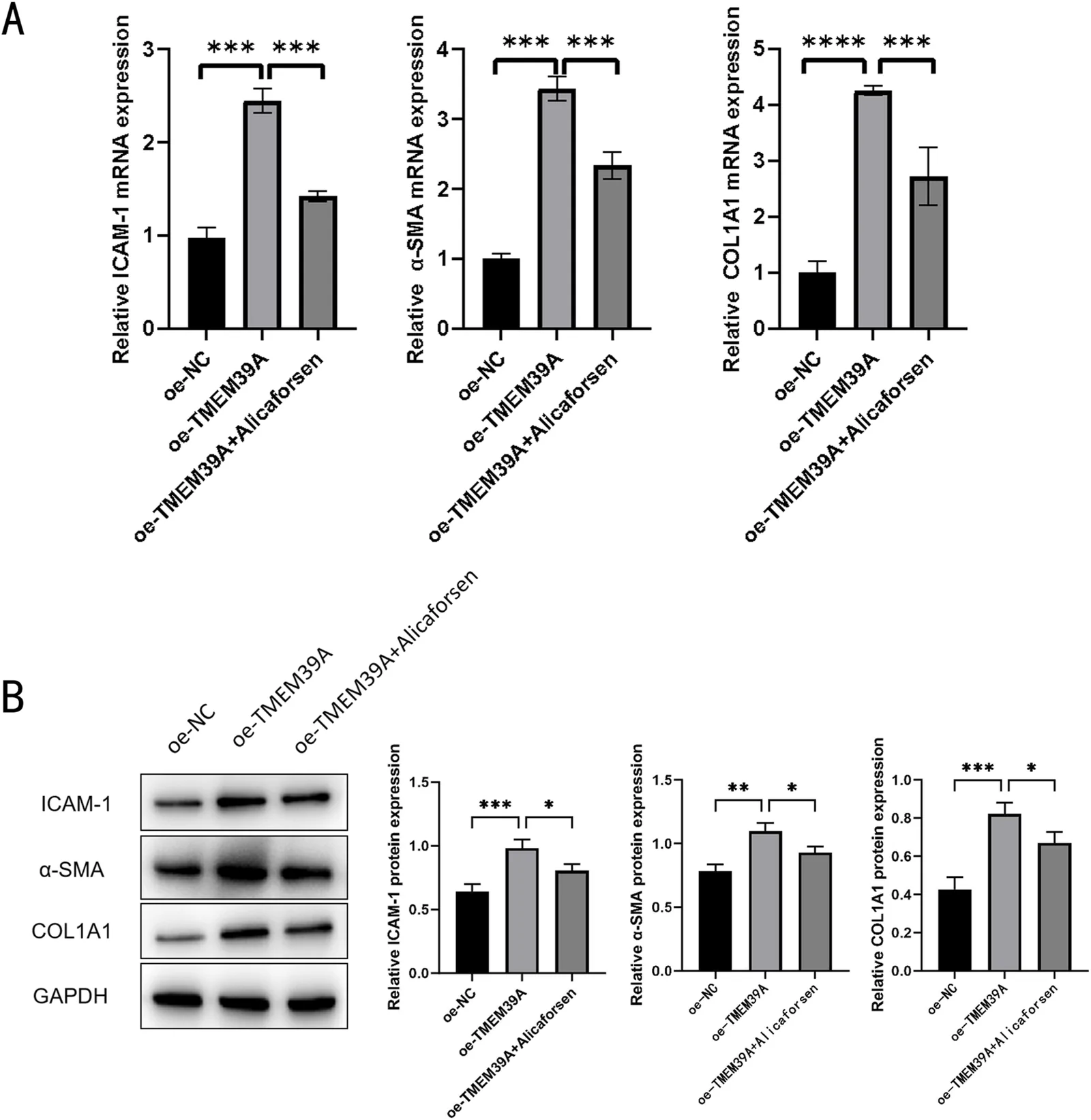
The CAM pathway inhibitor Alicaforsen partially reverses TMEM39A overexpression-induced upregulation of ICAM-1, α-SMA, and COL1A1. **(A)** RT-qPCR showing relative mRNA levels of ICAM-1, α-SMA, and COL1A1 in each group. **(B)** Western blotting showing protein levels of ICAM-1, α-SMA, and COL1A1 in each group. *P < 0.05, **P < 0.01, ***P < 0.001, ***P < 0.0001.

## 4 Discussion

The role of TMEM39A in liver fibrosis has remained unclear, with prior studies predominantly focusing on its functions in autophagy and immune regulation. GWAS have identified the 3q13.33 region harboring TMEM39A as a susceptibility locus for primary biliary cholangitis (Hitomi et al., 2019), and TMEM39A has also been linked to systemic lupus erythematosus and multiple sclerosis (Tran et al., 2017). Functionally, TMEM39A promotes encephalomyocarditis virus replication via autophagy (Li et al., 2019) and is highly expressed in glioma tissues (Park et al., 2017). However, its involvement in HSC activation has not been previously reported. Our study reveals, for the first time, that TMEM39A positively regulates TGF-β1-induced HSC activation: overexpression enhances α-SMA and COL1A1 expression, whereas knockdown suppresses them. Furthermore,our observation that TGF-β1 upregulates TMEM39A in a dose-dependent manner suggests that TMEM39A may function as a downstream responsive molecule of TGF-β1 signaling. TGF-β1 is known to activate Smad2/3 and non-Smad pathways (e.g., MAPK, PI3K/AKT) in HSCs (Shin et al., 2025; Oh et al., 2024), and it is plausible that TMEM39A transcription is directly or indirectly regulated by these cascades. However, whether Smad-binding elements or other transcription factors mediate this effect remains unknown. Future chromatin immunoprecipitation and promoter-reporter assays are warranted to delineate the regulatory relationship between TGF-β1 and TMEM39A.

Liver fibrosis results from various chronic injuries, with HSC activation and transdifferentiation into myofibroblasts as the core event (Filliol et al., 2022). During this process, hepatocytes, macrophages, and endothelial cells contribute via secreted cytokines and chemokines (Kisseleva and Brenner, 2021; Sun and Matsukawa, 2024). Our findings link TMEM39A—a previously uncharacterized molecule—to liver fibrosis, with a pro-fibrotic effect analogous to that of other known regulators. For example, CerS5 deficiency aggravates NAFLD-associated fibrosis (Chen et al., 2023), while Mboat7 loss promotes fibrosis through lipid signaling (Thangapandi et al., 2021). Conversely, Endoglin plays a protective role, and its deletion exacerbates fibrosis (Alsamman et al., 2018), whereas von Willebrand factor deficiency attenuates fibrosis (Joshi et al., 2017). These studies underscore the complexity of the fibrosis regulatory network, where different molecules exert distinct functions depending on cell type and microenvironment. Our results position TMEM39A as a novel pro-fibrotic regulator. Our results demonstrate that TMEM39A overexpression promotes LX-2 cell viability and migratory capacity while suppressing apoptosis, consistent with the activated HSC phenotype. Conversely, TMEM39A knockdown exerts opposite effects. These findings collectively establish TMEM39A as a positive regulator of HSC activation at the cellular functional level. However, it should be noted that although TMEM39A significantly reduced apoptosis, the very low baseline apoptosis rate in LX-2 cells suggests that the anti-apoptotic effect may be quantitatively minor under basal conditions. The pro-fibrotic phenotype of TMEM39A is more likely driven by enhanced proliferation and migration, as supported by CCK-8, wound healing, and Transwell assays.

By integrating transcriptomic profiling with functional rescue experiments, we constructed a comprehensive evidence chain from genome-wide expression exploration to mechanistic validation. GO analysis revealed enrichment of TMEM39A-induced DEGs in cell adhesion and cell-matrix adhesion processes, while KEGG analysis pointed to the CAM pathway. Previous studies have established that CAM pathway activation, particularly ICAM-1 involvement, is critical in liver inflammation and fibrosis. Activated HSCs upregulate ICAM-1 to enhance immune cell adhesion, exacerbating injury and fibrosis (Pu et al., 2024). GSEA further confirmed coordinated upregulation of gene sets related to cell adhesion molecule binding. These multi-layered analyses strongly suggest that the CAM pathway is a key downstream effector of TMEM39A’s pro-fibrotic action.

Cell adhesion molecules play crucial roles in HSC migration and inflammatory responses. ICAM-1, as a representative molecule, is considered a key event in HSC activation and fibrosis progression. Our study reveals a new function of TMEM39A as an upstream regulator of ICAM-1. Overexpression of TMEM39A upregulated ICAM-1, and pharmacological blockade with Alicaforsen reversed TMEM39A-induced HSC activation and fibrosis marker expression. This directly links TMEM39A to the classical CAM pathway, establishing a new node in the liver fibrosis regulatory network. The partial reversal by Alicaforsen indicates that while the CAM/ICAM-1 axis is a significant mediator of TMEM39A-driven HSC activation, it is likely not the sole effector. Our RNA-seq data also implicated MAPK signaling, adherens junction, and autophagy pathways, which may act in parallel or in crosstalk with CAM signaling. Future studies using pathway-specific inhibitors or CRISPR-based knockouts of multiple targets will be required to fully map the downstream network of TMEM39A.Given the established importance of cell adhesion molecules in mediating cell-cell interactions and inflammatory signaling during fibrosis, our findings provide a new perspective on HSC activation and identify TMEM39A as a potential upstream regulatory node. Integrating this node into the known molecular network broadens our understanding of HSC activation and offers a potential target for anti-fibrotic intervention.

Beyond transcriptional regulation, our findings raise the possibility that TMEM39A may also influence COL1A1 at the post-transcriptional or protein secretion level. TMEM39A is an ER-resident transmembrane protein, and its nematode ortholog TMEM-39 has been shown to coordinate with COPII vesicle complexes to regulate collagen secretion and ER stress responses (Zhang Z. et al., 2021). In mammalian cells, COPII-mediated transport is essential for proper secretion of procollagen, and disruption of this process leads to intracellular collagen accumulation and ER stress, which can secondarily upregulate fibrotic gene expression. Therefore, the increased COL1A1 levels observed in TMEM39A-overexpressing LX-2 cells may not solely result from enhanced transcription, but could also involve enhanced COPII-dependent collagen trafficking and secretion, or reduced ER-associated degradation. Conversely, TMEM39A knockdown might impair collagen secretion, leading to reduced extracellular matrix deposition. However, our current RT-qPCR and Western blot data primarily reflect total cellular COL1A1 levels, and we did not specifically distinguish between intracellular retention and secreted collagen. Future studies employing collagen secretion assays, pulse-chase labeling, and subcellular fractionation are warranted to determine whether TMEM39A modulates COL1A1 through transcriptional activation, post-translational processing, secretory trafficking, or a combination of these mechanisms. Such investigations would provide a more comprehensive understanding of how TMEM39A contributes to the fibrotic phenotype beyond its effects on HSC activation markers.

Furthermore, RNA-seq results suggest that TMEM39A may influence HSC function through multiple signaling pathways. TMEM39A (whose nematode ortholog TMEM-39) is an ER-localized transmembrane protein that coordinates with COPII vesicle complexes to regulate collagen secretion and vesicular transport (Zhang Z. et al., 2021). Enrichment analysis also pointed to the MAPK signaling pathway as a potential downstream effector. Numerous studies have confirmed the central role of MAPK—particularly p38 and ERK branches—in HSC activation and fibrosis. For example, conjugated bile acids activate p38 MAPK and ERK1/2 via TGR5, promoting fibrosis (Xie et al., 2021), and inhibiting p38 MAPK attenuates HSC activation and fibrosis (Liu et al., 2024). ERK2 signaling also regulates liver inflammation and fibrosis (Jeng et al., 2020). Natural products like kaempferol modulate macrophage M1 polarization via MAPK/NF-κB to alleviate fibrosis (Chen et al., 2025). Roxarsone inhibits HSC activation specifically through TGF-β1/Smad rather than MAPK or PI3K/AKT pathways (Li et al., 2023), while gemigliptin alleviates fibrosis by blocking TLR4/MAPK and activating SIRT1/AMPK/Nrf2 pathways (Alm et al., 2025). These studies illustrate the central role of MAPK and its crosstalk with other pathways. Future work should delineate the detailed downstream signaling network of TMEM39A through molecular and pharmacological approaches, systematically verifying whether TMEM39A mediates HSC proliferation, migration, and apoptosis via MAPK and related nodes, thereby fully elucidating its pro-fibrotic mechanism.

This study is the first to link TMEM39A to liver fibrosis pathogenesis, offering new insights into HSC activation mechanisms and potential intervention strategies. However, several limitations of this study should be acknowledged. First, all functional experiments were performed exclusively in the immortalized LX-2 cell line, which, while widely used as an *in vitro* model of HSC activation, may not fully recapitulate the biological characteristics of primary human hepatic stellate cells. Therefore, validation in primary HSCs isolated from human or murine livers, as well as in animal models of liver fibrosis such as CCl_4_-induced or bile duct ligation (BDL) models with cell-type-specific TMEM39A modulation, is essential to confirm the pathophysiological relevance and therapeutic potential of our findings. Second, our mechanistic evidence relies heavily on pharmacological inhibition of ICAM-1 using Alicaforsen, which may have off-target effects; genetic ablation of ICAM-1 via CRISPR/Cas9-mediated knockout or siRNA-mediated silencing is warranted to definitively establish the causal role of this adhesion molecule in TMEM39A-mediated HSC activation. Third, the upstream regulators of TMEM39A expression in HSCs remain completely unexplored. While we observed TGF-β1-induced upregulation of TMEM39A, the transcription factors, epigenetic modifications (e.g., DNA methylation, histone acetylation) (Page and Mann, 2015), or non-coding RNAs that govern TMEM39A expression during HSC activation have not been identified, and future studies should investigate whether similar regulatory mechanisms operate in this context. For instance, GRHL2 has been shown to activate MAPK via miR-200 upregulation, exacerbating NAFLD fibrosis (Wang et al., 2020), and TEAD4 regulates LPAR3 expression to influence HSC activation and portal hypertension (Qi et al., 2025). Moreover, macrophages release inflammatory and pro-fibrotic factors (Sun and Matsukawa, 2024; González Del Barrio et al., 2026), and hepatocyte lipotoxicity and apoptosis are key drivers of fibrosis progression (Engin, 2024). Exploring whether similar upstream signals or epigenetic modifications regulate TMEM39A expression will be an important direction for future research. Fourth, although RNA-seq implicated several additional pathways (e.g., MAPK, adherens junction, autophagy), we have not systematically dissected their contributions, and the crosstalk between these pathways and CAM signaling in the context of TMEM39A overexpression remains unclear. Given that both p38 MAPK and PI3K/AKT have been implicated in HSC activation and that the TGF-β1/Smad pathway serves as a core fibrotic cascade (Qi et al., 2025), and given that other molecules such as Osteopontin and Gas6 also regulate fibrosis through inflammation and immune responses (Nagoshi, 2014; Fourcot et al., 2011), subsequent studies using pathway-specific inhibitors and combinatorial approaches are needed to construct a complete signaling network downstream of TMEM39A. Collectively, before TMEM39A can be considered a clinically actionable target, a comprehensive body of evidence is required, encompassing not only the aforementioned *in vivo* and primary cell validations but also demonstration of TMEM39A dysregulation in human fibrotic liver tissues, rigorous assessment of off-target effects and isoform specificity, and thorough evaluation of therapeutic window and delivery strategies. Addressing these multifaceted limitations will be essential to translate our *in vitro* findings into future clinical applications.

## 5 Conclusion

This study is the first to demonstrate that TMEM39A acts as a novel positive regulator of HSC activation. TMEM39A overexpression significantly promotes HSC proliferation, migration, and fibrosis marker expression while inhibiting apoptosis. Mechanistically, these effects are mediated, at least in part, through activation of the CAM pathway involving ICAM-1. This discovery directly integrates TMEM39A into the molecular regulatory network of liver fibrosis, providing a new theoretical foundation and potential molecular target for therapeutic intervention.

## Data Availability

The datasets presented in this study can be found in online repositories. The names of the repository/repositories and accession number(s) can be found in the article/Supplementary Material.
