# Supplementary material for: TMEM39A acts as a novel profibrotic factor to activate hepatic stellate cells partially via the cell adhesion molecule signaling axis
Source: Front Cell Dev Biol. 2026 Sep 28;14:1926987. doi: 10.3389/fcell.2026.1926987 (PMC13656855; doi:10.3389/fcell.2026.1926987)
Supplement: Supplementary file 2 [file DataSheet2.pdf]

Figure S2

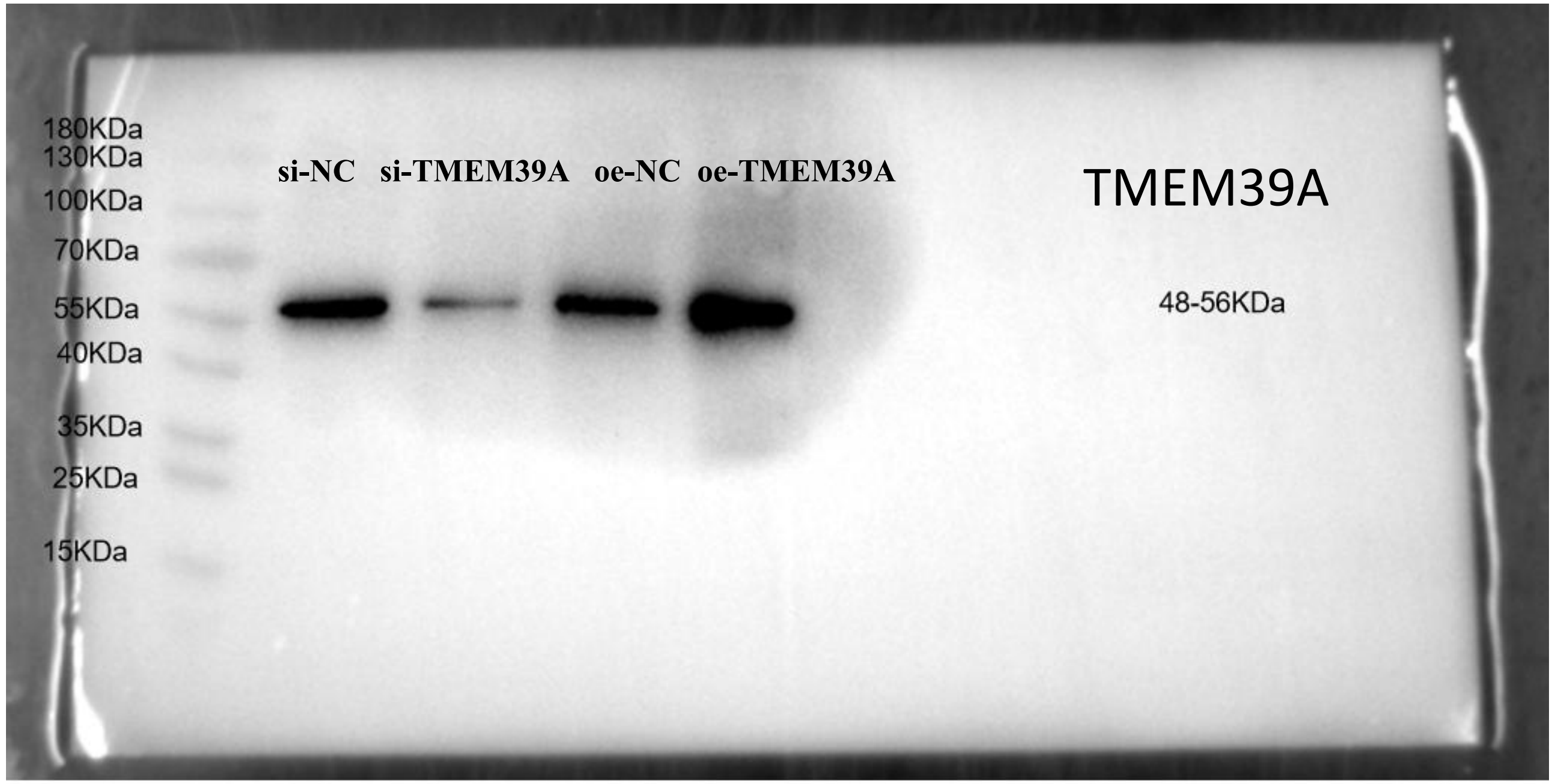

180KDa
130KDa
100KDa
70KDa
55KDa
40KDa
35KDa
25KDa
15KDa
si-NC
si-TMEM39A
oe-NC
oe-TMEM39A
TMEM39A
48-56KDa

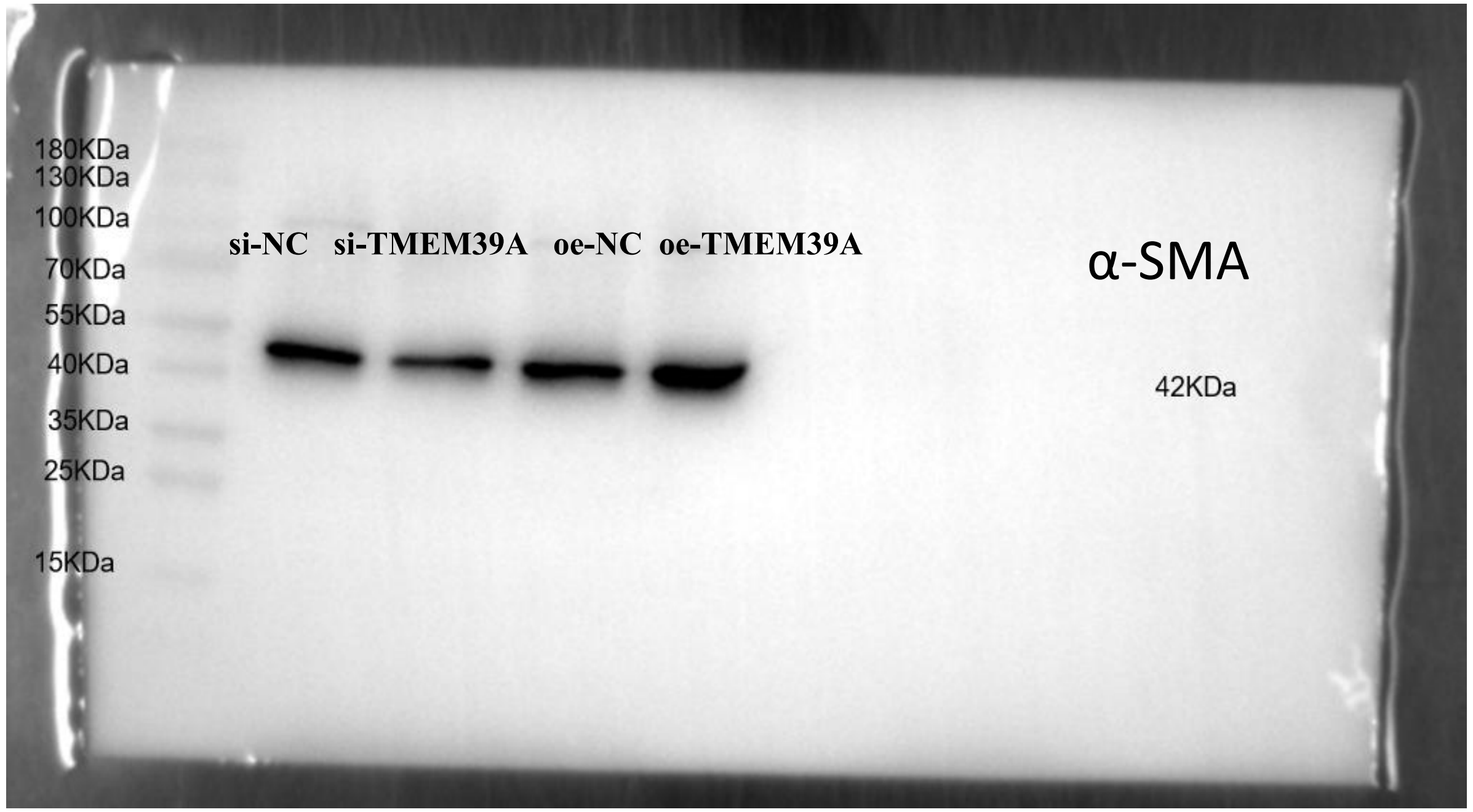

180KDa
130KDa
100KDa
70KDa
55KDa
40KDa
35KDa
25KDa
15KDa
si-NC
si-TMEM39A
oe-NC
oe-TMEM39A
α-SMA
42KDa

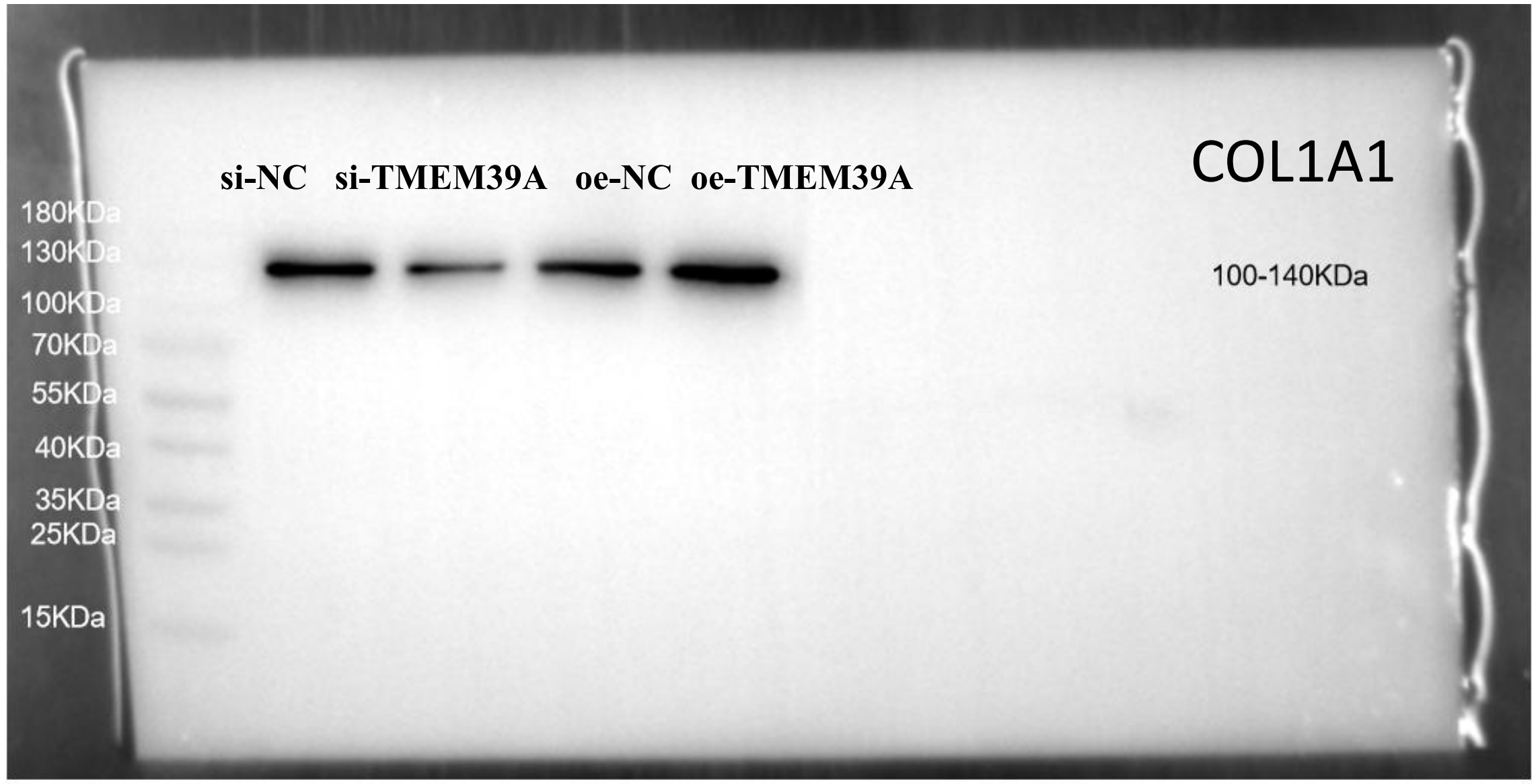
COL1A1
si-NC
si-TMEM39A
oe-NC
oe-TMEM39A
180KDa
130KDa
100KDa
70KDa
55KDa
40KDa
35KDa
25KDa
15KDa
100-140KDa

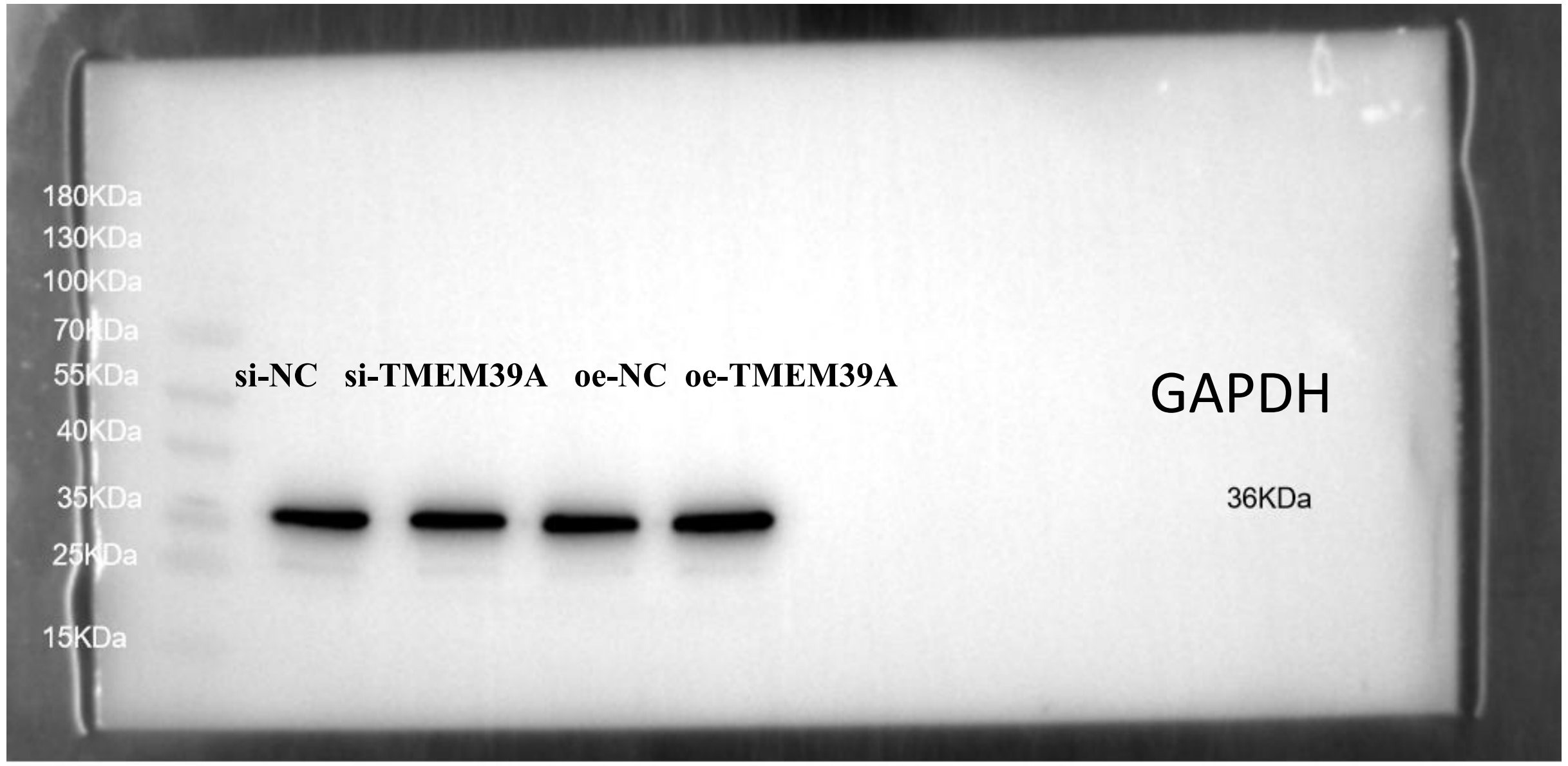
180KDa
130KDa
100KDa
70KDa
55KDa
40KDa
35KDa
25KDa
15KDa
si-NC
si-TMEM39A
oe-NC
oe-TMEM39A
GAPDH
36KDa

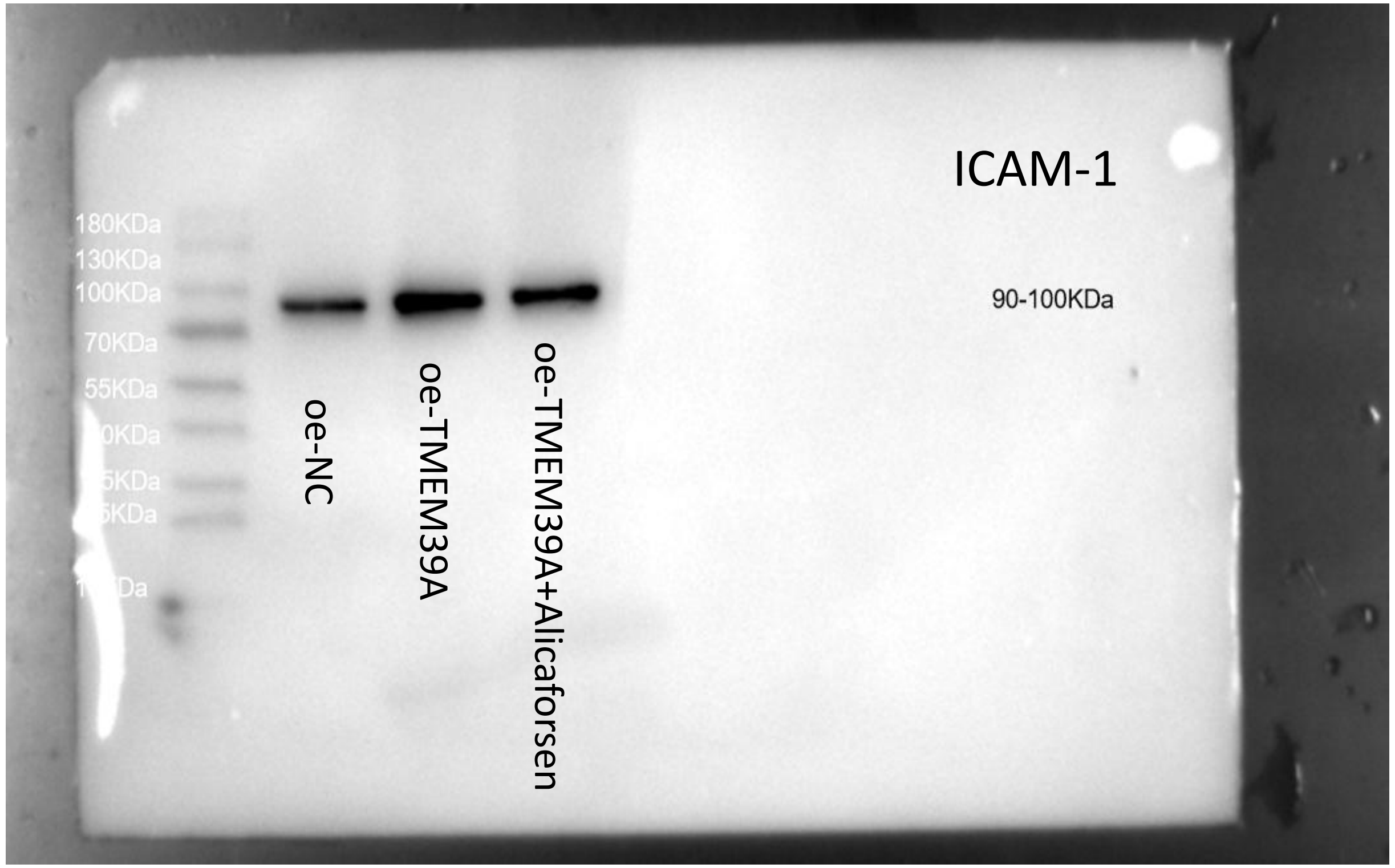
ICAM-1
180KDa
130KDa
100KDa
70KDa
55KDa
90-100KDa
oe-NC
oe-TMEM39A
oe-TMEM39A+Alicaforsen

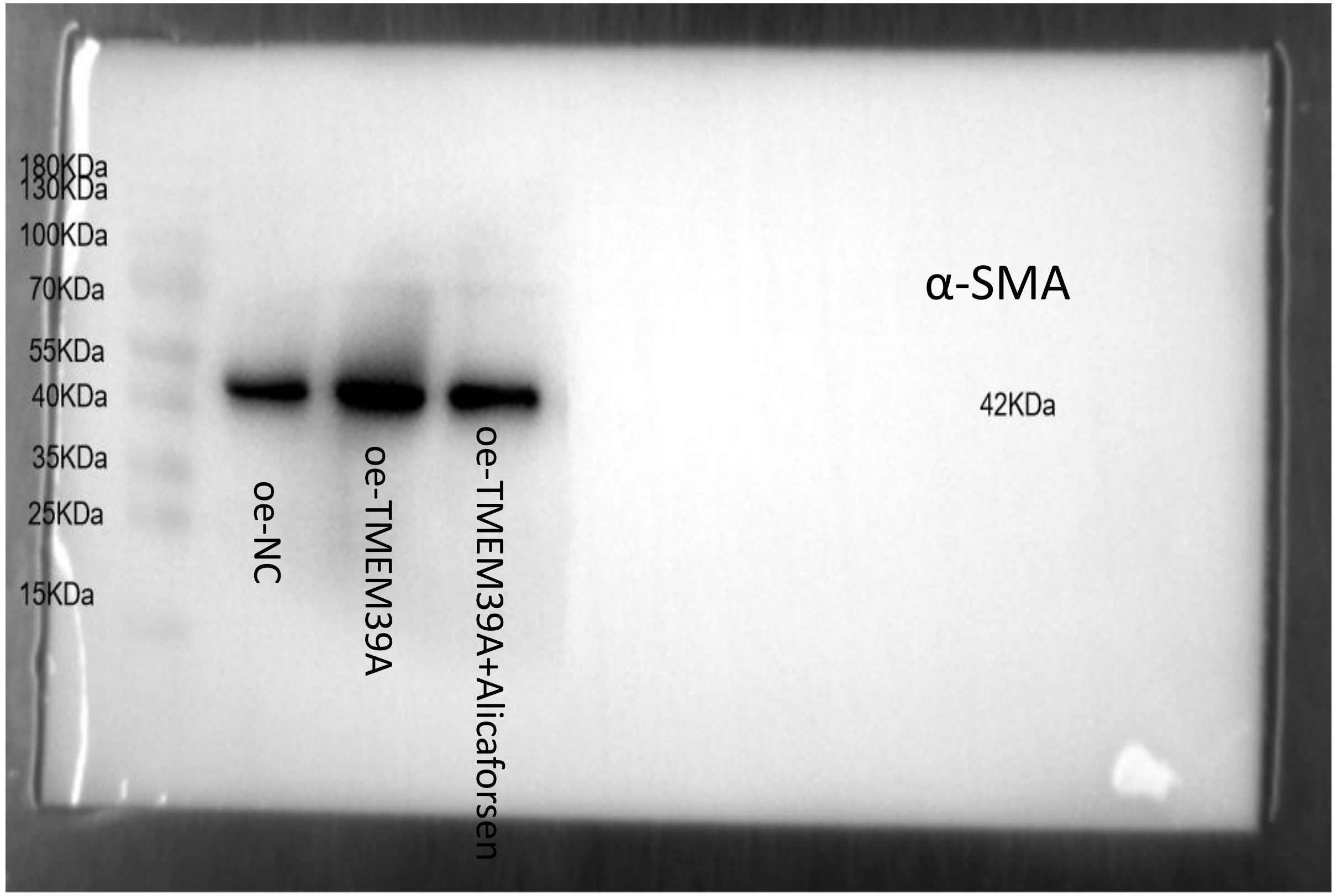
180KDa
130KDa
100KDa
70KDa
55KDa
40KDa
35KDa
25KDa
15KDa
α-SMA
42KDa
oe-NC
oe-TMEM39A
oe-TMEM39A+Alicaforsen

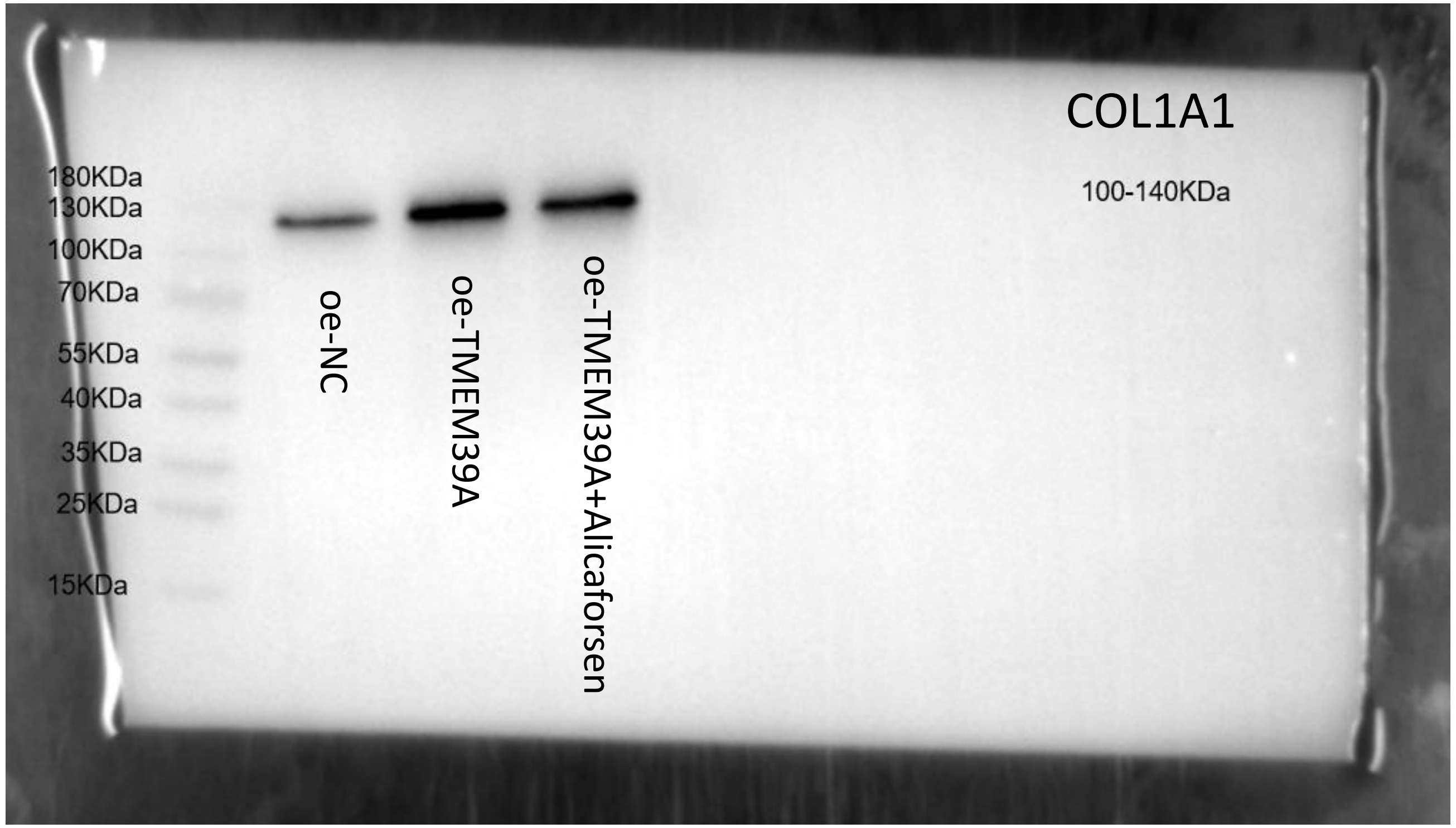
COL1A1
100-140KDa
180KDa
130KDa
100KDa
70KDa
55KDa
40KDa
35KDa
25KDa
15KDa
oe-NC
oe-TMEM39A
oe-TMEM39A+Alicaforsen

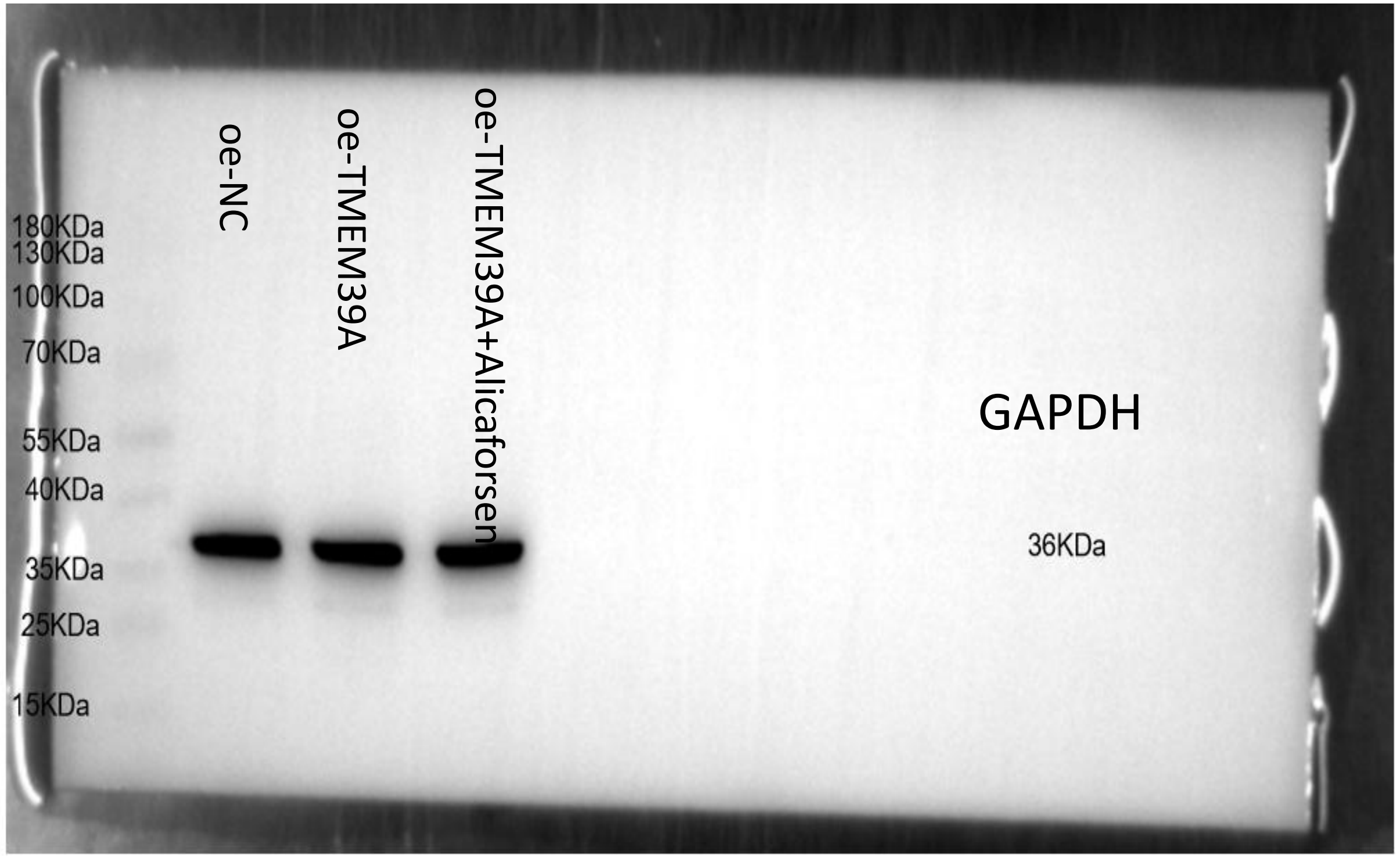
oe-NC
oe-TMEM39A
oe-TMEM39A+Alicaforsen
GAPDH
36KDa
180KDa
130KDa
100KDa
70KDa
55KDa
40KDa
35KDa
25KDa
15KDa
