# Supplementary material for: TMEM39A acts as a novel profibrotic factor to activate hepatic stellate cells partially via the cell adhesion molecule signaling axis
Source: Front Cell Dev Biol. 2026 Sep 28;14:1926987. doi: 10.3389/fcell.2026.1926987 (PMC13656855; doi:10.3389/fcell.2026.1926987)
Supplement: Supplementary file 3 [file DataSheet3.pdf]

Figure S3: The clustering diagram of PCA (Principal Component Analysis) for the gene expression levels of the samples; the abscissa represents the dimension of the first principal component, and the ordinate represents the dimension of the second principal component.

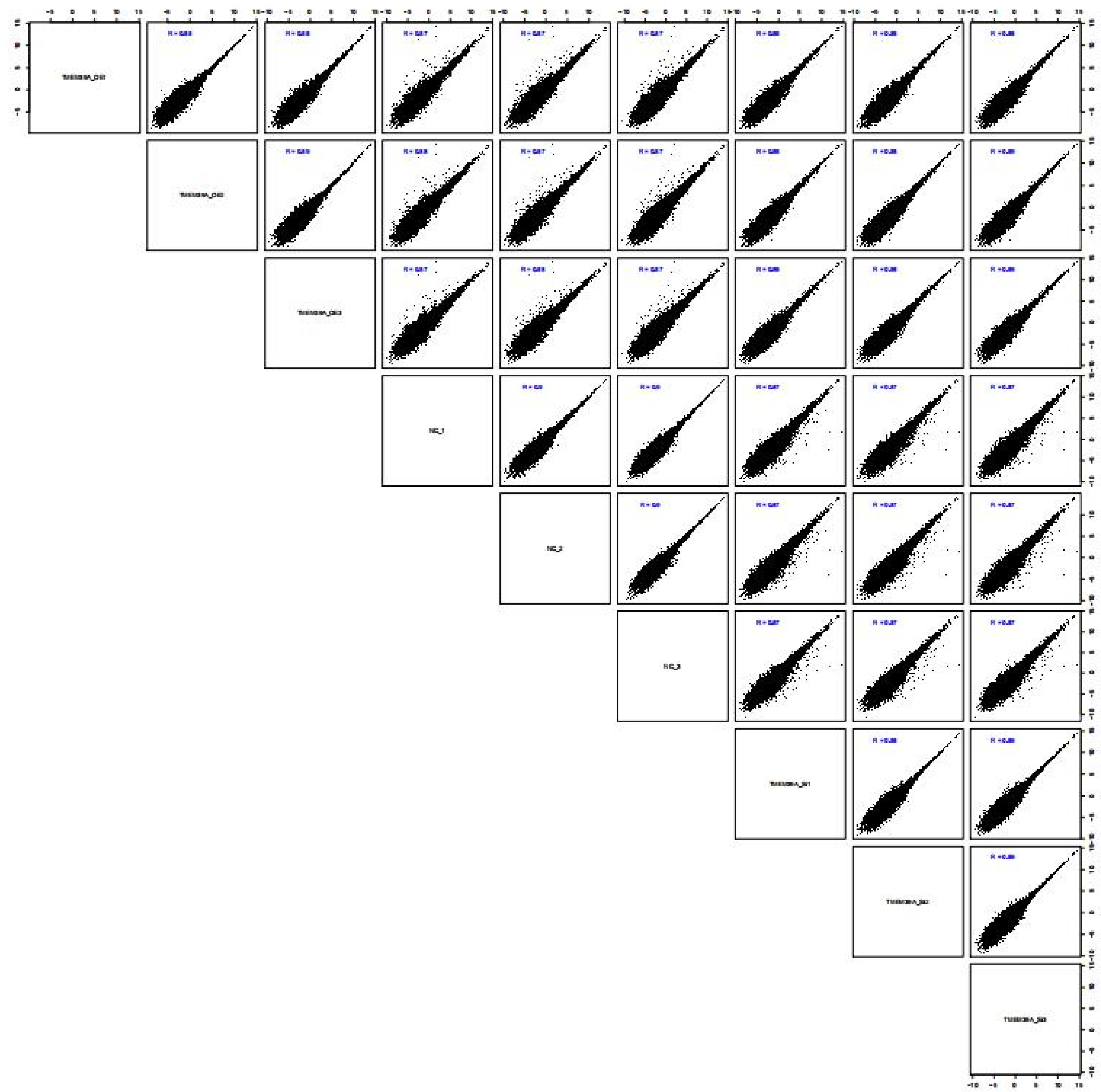

Sample_RPKM_correlation
