# Supplementary material for: TMEM39A acts as a novel profibrotic factor to activate hepatic stellate cells partially via the cell adhesion molecule signaling axis
Source: Front Cell Dev Biol. 2026 Sep 28;14:1926987. doi: 10.3389/fcell.2026.1926987 (PMC13656855; doi:10.3389/fcell.2026.1926987)
Supplement: Supplementary file 4 [file DataSheet4.pdf]

Figure S4 shows the scatter plot of gene expression correlations among the samples; the x-axis and y-axis represent the log10(RPKM) values of genes in different samples respectively.

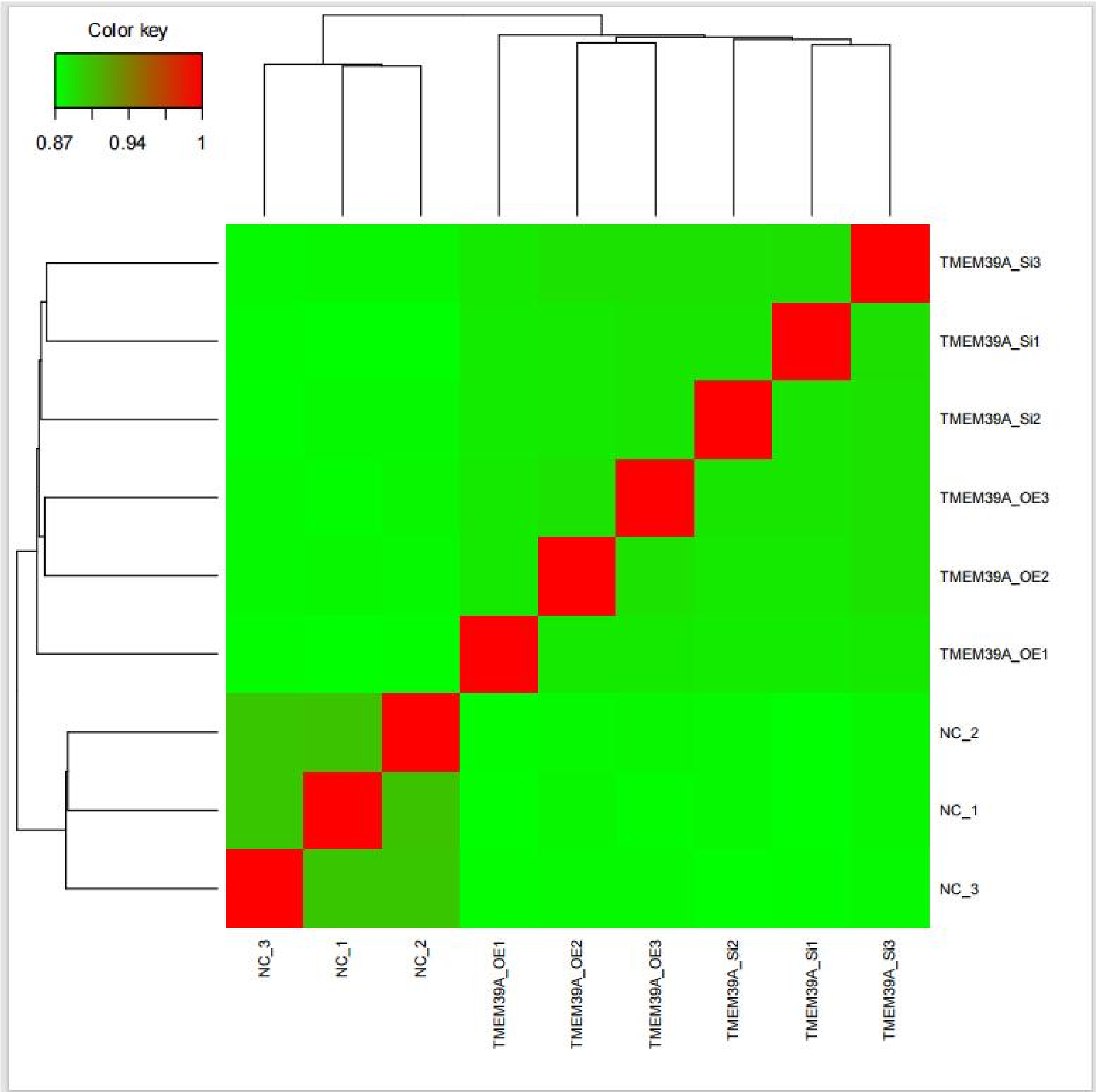

Sample_corr_heatmap
