# Supplementary material for: TMEM39A acts as a novel profibrotic factor to activate hepatic stellate cells partially via the cell adhesion molecule signaling axis
Source: Front Cell Dev Biol. 2026 Sep 28;14:1926987. doi: 10.3389/fcell.2026.1926987 (PMC13656855; doi:10.3389/fcell.2026.1926987)
Supplement: Supplementary file 5 [file DataSheet5.pdf]

Figure S5 Hierarchical clustering diagram of sample gene expression levels; the color scale ranges from green to red, representing the lower inter-sample correlation obtained through gene expression levels and the higher inter-sample correlation. The branch segments generated from the same node represent that the corresponding samples can be clustered into one group, and the length of the branch represents the similarity of the samples: the shorter the length, the higher the similarity between the samples; the longer the length, the lower the similarity between the samples.

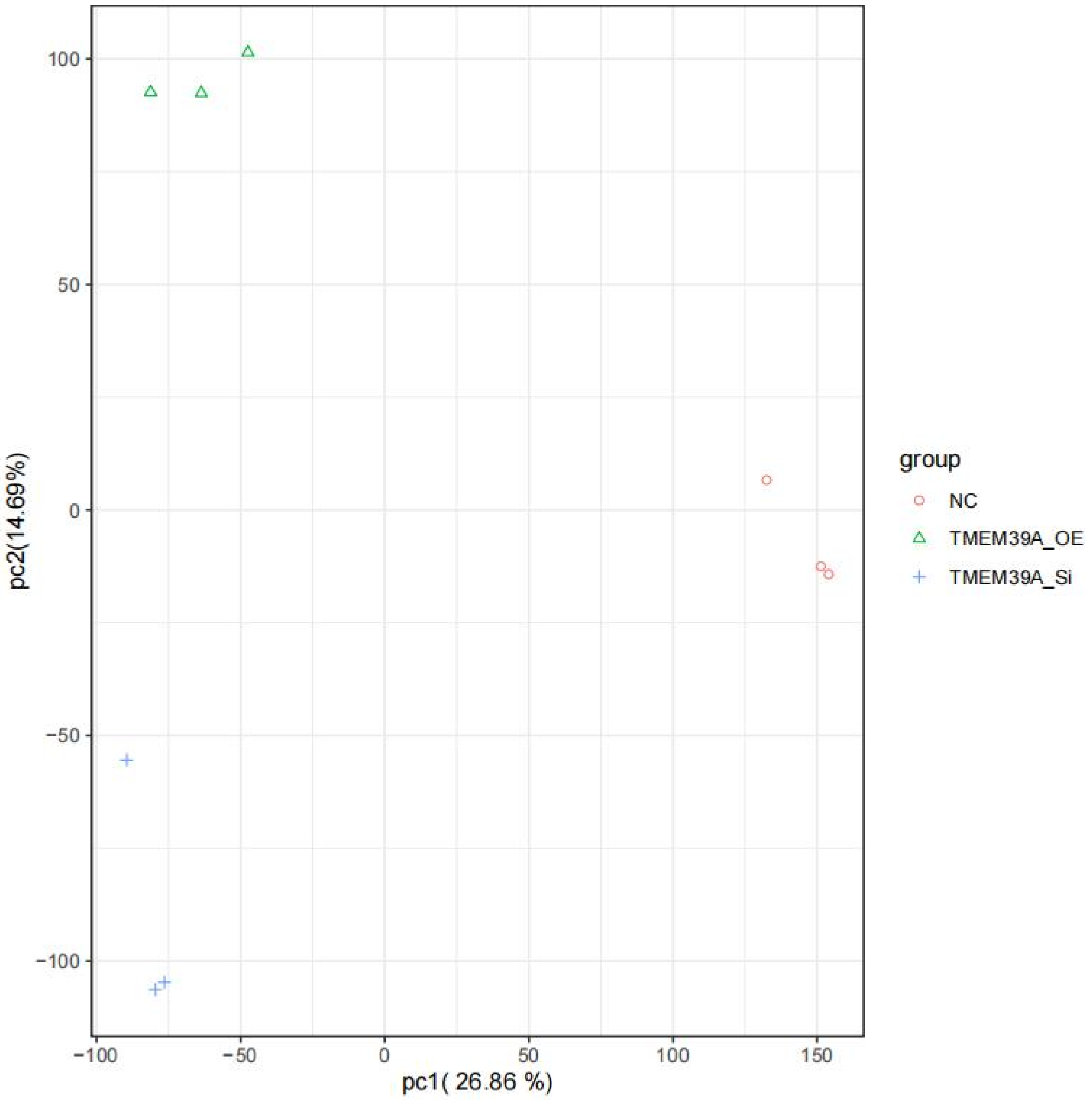

Sample_PCA_correlation
