# Supplementary material for: TMEM39A acts as a novel profibrotic factor to activate hepatic stellate cells partially via the cell adhesion molecule signaling axis
Source: Front Cell Dev Biol. 2026 Sep 28;14:1926987. doi: 10.3389/fcell.2026.1926987 (PMC13656855; doi:10.3389/fcell.2026.1926987)
Supplement: Supplementary file 6 [file Table1.docx]

Table 1. Gene primer sequence.

| **Gene** | **Sequence（5’-3’）** |
| --- | --- |
| TMEM39A-F | CCCACCTATCACAGCCTTAATC |
| TMEM39A-R | AAAGAGCAACCAACAGGTAGAT |
| ICAM-1-F | AGCGGCTGACGTGTGCAGTAAT |
| ICAM-1-R | TCTGAGACCTCTGGCTTCGTCA |
| COL1A1-F | CGATGGATTCCAGTTCGAGT |
| COL1A1-R | TTTTGAGGGGGTTCAGTTTG |
| ACTA2-F | ACTGAGCGTGGCTATTCCTCCGTT |
| ACTA2-R | GCAGTGGCCATCTCATTTTCA |
| GAPDH-F | GGTGGTCTCCTCTGACTTCAACA |
| GAPDH-R | TTTGCTGTAGCCAAATTCGTTGT |
| si-TMEM39A-F | GGUGGUAUCCUUACAAUCA |
| si-TMEM39A-R | UGAUUGUAAGGAUACCACC |
| si-NC-F | UUCUCCGAACGUGUCACGUTT |
| si-NC-R | ACGUGACACGUUCGGAGAATT |
